# Assessment of association between smoking and all-cause mortality among Malaysian adult population: Findings from a retrospective cohort study

**DOI:** 10.18332/tid/147656

**Published:** 2022-05-31

**Authors:** Kuang Hock Lim, Yoon Ling Cheong, Hui Li Lim, Chee Cheong Kee, Sumarni Mohd Ghazali, Balvinder Singh Gill Pradmahan Singh, Mohd Azahadi Omar, Mohd Hazilas Mat Hashim, Yong Kang Cheah, Jia Hui Lim

**Affiliations:** 1 Institute for Medical Research, Setia Alam, Malaysia; 2 Institute for Medical Research, Kuala Lumpur, Malaysia; 3 Clinical Research Centre, Hospital Sultan Ismail, Johor Bahru, Malaysia; 4 Department of Biostatistics and Data Repository, National Institutes of Health, Shah Alam, Malaysia; 5 School of Economics, Finance and Banking, Universiti Utara Malaysia, Sintok, Malaysia; 6 Pharmacy Department, Hospital Putrajaya, Putrajaya, Malaysia

**Keywords:** smoking, all-cause mortality, Malaysian adults

## Abstract

**INTRODUCTION:**

Smoking is a known risk factor for many chronic diseases. Illness and death due to smoking are a significant public health burden in many countries. This study aims to address the information gap in smoking-related mortality in Malaysia by estimating the risk of cardiovascular disease and all-cause mortalities due to smoking among Malaysian adults.

**METHODS:**

We analyzed data on 2525 respondents, aged 24–64 years, of the Malaysian Non-Communicable Disease Surveillance survey conducted September 2005 to February 2006. Mortality records from the Malaysian National Registration Department were linked to the MYNCDS-1 data to determine respondents’ mortality status over 12 years from 2006 to 2018. Associations between smoking and all-cause mortalities were assessed using Cox proportional hazards regression with adjustments for non-communicable disease and sociodemographic and lifestyle factors.

**RESULTS:**

The prevalence of daily smoking was 21.2% (95% CI: 19.0–23.7). During the 31668 person-years follow-up, 213 deaths from all causes occurred, where 68 deaths were among smokers (13.2%), and 452 were among non-smokers (6.3%). Smoking was associated with a significantly increased risk of all-cause mortality (adjusted hazard ration, AHR=1.79; 95% CI: 1.12– 2.97). These associations remained significant after excluding mortalities in the first two years of follow-up.

**CONCLUSIONS:**

Daily smoking is associated with a significantly higher risk of all-cause death. Behavioral and pharmacological smoking cessation interventions should be intensified among smokers to reduce the risk of mortality.

## INTRODUCTION

Tobacco smoking is the single major cause of premature death globally^1^, killing more than 8 million people a year around the world^2^. More than 7 million of these deaths are the result of direct tobacco use^2^. Despite substantial reductions in tobacco use^3^, smoking is still the leading cause of preventable disease worldwide^4^, accounting for 200 million disability-adjusted life-years, and is the leading risk factor for death among males (20.2% of male deaths)^4^. In addition, tobacco smoking is an established cause of cancer^5,6^, and increases the risk for cardiovascular and chronic respiratory diseases. A rise in the smoking rate is followed 10–20 years later by an increase in the incidence of diseases such as ischemic heart disease, lung, oral cavity and larynx cancers; and 20–40 years later, by chronic obstructive airway disease^5,6^.

In Malaysia, it is estimated that 20000 deaths attributed to smoking occur each year. Smoking-related diseases have been identified as a significant contributor to disability-adjusted life years and years of life lost among the Malaysian population^7^. In addition, 2.9 billion MYR (100 Malaysian Ringgit about 23 US$) were spent on treating chronic obstructive pulmonary disease (COPD), lung cancer and ischemic heart disease (IHD), three diseases related to smoking; this is equivalent to 0.6% of the country’s GDP or 16.5% of the national health expenditure^8^. More than one-fifth of Malaysian adults are current smokers, and this prevalence has plateaued for the past three decades^9-13^. The Ministry of Health, Malaysia, has initiated and implemented several anti-smoking policies and measures to reduce the prevalence of smoking among the Malaysian population from 22% to 15% by 2025^9^. More proactive and innovative measures should be formulated to address the high smoking prevalence among Malaysian adults. According to the Health Belief Model, people are more likely to change their behavior if given information that they can relate to similar lifestyles, beliefs, and social and cultural backgrounds^14^. Evidence on the risk of mortality due to smoking derived from local data will add credibility to smoking intervention efforts and will be more likely to influence the local population than data from other countries, given the different environmental conditions such as socioeconomic factors, stress and genetic factors between Malaysia and those countries. Thus, locally-derived evidence on the magnitude of the health risks and disease burden due to smoking is particularly important for policymakers in planning preventive strategies and improving the perception of the risk of premature death due to smoking among the general population.

The impact of tobacco on society can be measured in terms of its mortality burden or tobacco-attributable deaths^15^. The World Health Organization reported that 11.5% of global deaths (6.4 million) in 2015 were attributable to smoking, of which 52.2% took place in four countries (China, India, the USA, and Russia). Smoking ranks among the five leading risk factors by disability-adjusted life years (DALYs) in 109 countries and territories in 2015, increasing from 88 countries in 1990^15^. An estimated 11% of all cardiovascular deaths in the world in 2000 could be attributed to tobacco-related diseases, and more specifically to ischemic heart disease and cerebrovascular disease^16^. Many studies have revealed that smoking increases all-cause mortality rates. Tahfouti et al.^17^ found that 9.7% of deaths among people aged >35 years in the Casablanca region, Morocco, were due to smoking. In other reports, Gu et al.^18^ revealed that 673000 deaths in 2005 in China were attributed to smoking. In the 16 Brazilian regions, 24222 (13.63%) deaths of people aged ≥35 years in 2003 were related to smoking^19^. Similarly, meta-analysis of pooled data from Asian countries (China, Japan, India, South Korea, Singapore and Taiwan) showed all-cause mortality in association with current smoking in successive birth cohorts, for the periods pre-1920, the 1920s, and 1930 or later, had hazard ratios (HRs) of 1.26 (95% CI: 1.17–1.37), 1.47 (95% CI: 1.35–1.61) and 1.70 (95% CI: 1.57–1.84), respectively^20^. In addition, Holipah et al.^21^ also reported similar findings, with an adjusted HR of all-cause death of 1.48 (95% CI: 1.11–1.98). However, less is known regarding the link between smoking prevalence and mortality in Malaysia.

To date, there have been no reports on the association of smoking with all-cause death in Malaysia. In common with many countries, Malaysia has relied on the findings from studies conducted in developed countries such as Australia^22^, the United Kingdom^23^, the USA^24^, and Asian countries^20^ to assess the impact of smoking on the Malaysian population. However, the effect of smoking on mortality in the population is related to the previous and current prevalence of smoking and the duration and intensity of smoking. These parameters are influenced by the epidemiology of smoking, the population investigated, the effectiveness of anti-smoking policies implemented, and social, economic and cultural factors. Therefore, the impact of smoking on mortality among the population may differ from country to country.

In view of these factors, data from Malaysia on the effect of smoking on mortality is needed for policymakers in formulating suitable policies with regard to smoking. Malaysian health care practitioners would be able to use these findings as local evidence of the harmful effects of tobacco in their health promotion activities. In addition, the information would also add to the existing evidence on this topic by providing data from a developing country plagued by smoking-related diseases for the past three decades. This study aimed to investigate whether smoking is associated with all-cause mortality in the Malaysian adult population.

## METHODS

### Study design

We derived data from the Malaysian Non-Communicable Disease Surveillance System 1 (MyNCDS-1) survey. MyNCDS-1 was a population-based survey on non-communicable diseases and their risk factors conducted September 2005 to February 2006. A representative sample of Malaysian adults was selected using the Department of Statistics Malaysia year 2000 national household sampling frame through multistage, cluster sampling. The stratifying variables were state and setting (urban/rural), with enumeration blocks, and living quarters and households as the primary, secondary, and elementary sampling units. Sample size by state was proportionate to the size of the population of each state. In all, 398 enumeration blocks and 1683 living quarters were selected. All eligible household members, aged 25–64 years, in the selected living quarters were invited to participate in the study except for pregnant women, severely ill, mentally unfit, and institutionalized individuals. Data collection was carried out from September 2005 to February 2006, with 2572 adults participating in the survey. The details of the methodology and results of MyNCDS-1 have been described elsewhere^25^.

### Baseline data

Baseline data extracted from the MyNCDS-1 2005/2006 survey were participants’ sociodemographic characteristics (i.e. gender, ethnicity and age), lifestyle factors (i.e. physical activity, smoking status and alcohol consumption), anthropomorphic measurements (i.e. height, weight and waist circumference), blood pressure and biochemical measurements (i.e. lipid and glucose profile).

### Assessment of smoking status

Smoking status was classified into two groups, smoker and non-smoker. Smokers were defined as smoking any tobacco product daily at least once a day (e.g. daily smoker), or smoked but not every day (e.g. occasional/non-daily smoker) at the time of the survey. Non-smokers were those who had never smoked or had ceased smoking for more than six months.

### Assessment of lifestyle risk factors and non-communicable diseases

Two lifestyle risk factors were included as covariates: current drinking and physical activity. Current drinking was defined as consumption of any amount of alcohol in the past 12 months. The Global Physical Activity Questionnaire version 2.0 was used to measure physical activity. Based on the IPAQ scoring protocol, the physical activity level and intensity were calculated in metabolic equivalent task minutes per week (MET-min/week)^26^. Persons with a Physical Activity score of at least 600 MET-min/week were considered as being physically active. Participants with average systolic blood pressure ≥140 mmHg and/or diastolic pressure ≥90 mmHg or known case of hypertension^27^ or have been told that they have hypertension by a doctor or medical assistant were classified as having hypertension. While respondents who answered yes to an item that they are being told they have diabetes by a doctor or medical assistant, and those with no known diabetes, were screened for diabetes mellitus using the two-hour postprandial glucose tolerance test. Those with a fasting plasma glucose level ≥7.0 mmol/L^28^ or were known cases of diabetes mellitus were classified as having diabetes mellitus. In addition, participants were classified as having hypercholesterolemia if their total cholesterol was >5.2 mmol/L or if they were previously diagnosed with dyslipidemia or hypercholesterolemia.

### Follow-up

Participants of MyNCDS-1 survival status were followed-up for approximately 13 years from March 2006 to December 2018. Mortality data during this period were obtained by matching the survey participants’ identification numbers with records in the death registry administered by the National Registration Department of Malaysia.

### Statistical analysis

The statistical analyses were performed using IBM SPSS version 29.0 with complex samples add-on module (IBM Corp., Armonk, New York, USA). All of the analyses took into account the complex survey design and unequal selection probabilities. The prevalence of smoking, sociodemographics and other lifestyle risk factors are presented as frequencies, percentages and 95% confidence intervals (CI) by survival status (dead/alive). The Kaplan-Meier analysis was carried out to describe the survival rate between smokers and non-smokers. The log rank test was applied to test for statistically significant differences in survival rates between smokers and non-smokers. The multivariable analysis by Cox regression was carried out to determine the association between smoking status and all-cause mortality. Three Cox proportional-hazards regression models were used to determine the association between smoking and all-cause mortality, adjusting for age (Model 1), adjusting for age and gender (Model 2), and adjusting of all independent variables (selected sociodemographic characteristics and health risk behaviors) (Model 3). For sensitivity analyses, Cox-regression models were also generated for all-cause mortality, excluding mortalities in the first two years of follow-up. The proportional hazards test was performed for all models, and all models met the assumption (p>0.05). Two-way interactions between all independent variables in each model were explored, the p>0.05 indicating there were no significant two-way interactions. In addition, *post hoc* power of the study was also calculated using PS Power software. Given a value of alpha of 0.05, frequency of smokers and non-smokers of 448 and 2077, respectively, accrual interval of 1 year, and additional follow-up after the accrual of 13 years, median survival time of the non-smoking group of 6.42 years (using the formula: median=t×log_e_(1/2)/log_e_(p), where p is the probability of survival among non-smokers until time t) and hazard ratio of 1.79, the power of the study was >90%.

## RESULTS

Of the 2572 participants of MyNCDS-1, a total of 2525 participants were included in the final sample after excluding participants with missing data on identification number (98.2% response rate). Forty-seven participants were excluded due to incomplete identification numbers. The final sample consisted of 1013 (40.1%) men and 1512 (59.9%) women. Among the respondents, more than half were of Malay ethnicity (n=1397; 55.4%), and attained secondary school level of education (n=1285; 54.3%). More than one-third of the respondents aged 25–34 years and 124% (n=225) consumed alcohol at least once in the last year. In addition, almost a third of respondents had diabetes and hypertension (either known or through biochemical testing). Nearly half of the respondents had hypocholesterolemia. The prevalence of smoking at baseline was 21.1%, and it was significantly higher among males compared to females (39.1% vs 2.1%), Malay and other ethnic groups with primary and secondary education level, and among those who consumed alcohol (42.2% vs 18.6%, p<0.001). The detailed sociodemographic characteristics, the prevalence of smoking and the prevalence of health risk behaviors of the participants, are shown in Table 1.

**Table 1:** Characteristics of participants and smokers (N=2525)

*Characteristics*	*Estimated population*	*Participants*		*Smokers*	
		*n*	*%*	*%*	*95 % CI*
**Smoking status**					
Smoker	2483462	448	21.1		
Non-smoker	9282234	2077	78.9		
**Gender**					
Male	6048468	1013	51.4	39.1	35.0–43.3
Female	5717228	1512	49.6	2.1	1.4–3.0
**Ethnicity**					
Malay	6519787	1397	55.4	22.0	18.8–25.6
Chinese	2049143	461	17.4	17.3	13.0–23.0
Indian	1131231	230	9.6	14.9	8.5–24.9
Other	2065534	437	17.6	25.4	20.9–30.9
**Age** (years)					
25–34	4152132	586	35.3	20.1	16.3–24.6
35–44	3529892	721	30.0	24.1	19.5–29.4
45–54	2609464	732	22.2	19.7	15.9–24.2
55–64	1474208	476	12.8	19.1	15.1–24.0
**Education level**					
≤Primary school	3954763	1023	33.6	21.7	18.2–25.6
Secondary school	6418097	1285	54.3	22.2	19.0–25.7
Tertiary education	1392836	217	11.8	14.6	8.9–23.3
**High fasting blood glucose or known DM type 2**					
Yes	3856873	818	32.8	18.8	15.1–22.2
No	7908823	1707	67.2	22.2	19.5–24.2
**High blood pressure or known hypertension**					
Yes	4109806	1047	34.9	20.3	17.2–23.8
No	7655890	1479	65.1	21.5	18.5–24.9
**Hypertriglyceridemia**					
Yes	6003601	1424	51.0	20.0	17.1–23.2
No	5762095	1101	49.0	22.3	18.9–26.1
**Physical active**					
Active	6931243	1352	58.9	23.0	20.0–26.3
Inactive	4834454	1173	41.1	17.2	14.2–20.6
**Alcohol drinker**					
Yes	1458580	252	12.4	42.4	35.0–50.3
No	10307116	2273	87.6	18.1	15.8–20.6

Table 2 shows that during the 13 years of follow-up, a total of 213 deaths [68 (13.2%) in the smoking group and 145 (6.3%) in the non-smoking group] from all causes were recorded by the National Registration Department, with a total follow-up time of 31668 person-years. A 2-fold or higher mortality among smokers compared to non-smokers was observed in most independent variables (e.g. ethnicity).

**Table 2:** Mortality rates in the study cohort during follow-up by smoking status and independent variables

*Variables*	*Smokers*				*Non-smokers*			
	*Estimated population*	*n*	*%*	*95% CI*	*Estimated population*	*n*	*%*	*95 % CI*
**Overall**	326617	68	13.2	9.2–18.5	581267	145	6.3	5.0–7.9
**Gender**								
Male	318550	65	13.5	9.2–19.1	344064	61	9.3	6.6–13.1
Female	8066	3	6.8	2.0–20.8	237023	84	4.2	3.3–5.5
**Ethnicity**								
Malay	204798	45	14.3	9.0–21.9	328224	78	6.5	4.6–9.0
Chinese	60334	7	16.9	6.0–39.1	107567	26	6.4	3.9–10.2
Indian	20992	5	12.4	3.9–32.9	56258	15	5.8	2.9–11.4
Other	40492	11	7.7	4.1–14.0	89216	26	5.8	3.8–8.7
**Age** (years)								
25–34	113719	7	13.6	5.7–28.9	66374	13	2.0	1.0–3.9
35–44	23051	5	2.7	1.1–6.7	80272	19	3.0	1.7–5.2
45–54	96836	27	18.4	12.4–27.5	177680	49	8.5	5.5–12.8
55–64	93009	29	33.0	21.3–47.2	256939	64	21.6	15.7–28.9
**Education level**								
≤Primary school	170955	42	20.0	13.0–29.4	297773	87	9.6	7.3–12.5
Secondary school	149953	25	10.5	5.7–18.6	263033	53	5.9	3.5–7.8
Tertiary education	5706	1	1.7	0.2–11.6	20459	5	1.7	0.8–4.8
**High fasting blood glucose or known DM type 2**								
Yes	248864	42	14.2	9.0–21.5	305497	68	5.0	3.5–7.0
No	77753	26	10.7	6.7–16.6	275770	77	8.8	6.5–11.9
**High blood pressure or known hypertension**								
Yes	164165	49	19.7	14.2–26.2	427579	102	13.1	10.0–16.6
No	162452	19	9.9	5.1–18.2	153687	43	2.0	1.7–3.8
**Hypertriglyceridemia**								
Yes	158466	41	13.2	8.0–19.1	344055	88	7.2	5.2–9.7
No	168151	27	13.1	7.2–22.7	237211	57	5.3	3.8–7.4
**Physical activity**								
Physically active	253532	49	13.9	8.9–20.9	361109	77	5.9	4.2–8.2
Physically inactive	73085	19	11.2	8.6–18.3	220157	68	7.0	5.4–9.1
**Alcohol drinker**								
Yes	71471	13	11.6	5.0–24.5	391721	9	4.7	2.3–9.3
No	255146	55	13.7	9.2–19.9	542095	136	6.4	5.0–8.1

**Figure 1:**
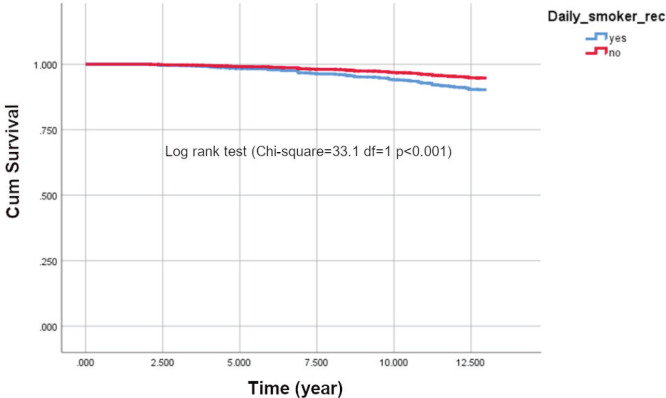
Kaplan Meier survival curves for smokers and non-smokers

The survival function (Figure 1) shows that cumulative survival after the follow-up period of 13 years was higher among non-smokers. The survival rates of the two groups started to differ after five years, and the trend continued until the end of the observation period (log rank test χ^2^=33.1, df=1, p<0.001).

The multivariable analysis revealed that smoking is significantly associated with an increased risk of all-cause mortality after adjusting for age (adjusted hazard ratio, AHR=2.42; 95% CI: 1.55–3.76). Sensitivity analysis showed that the association remained significant after excluding the first two years of mortalities in the model, adjusted for age and gender (AHR=1.81; 95% CI: 1.10–3.02), and in the final model adjusted for all independent variables (AHR=1.83; 95% CI:1.11–3.02) (Table 3).

**Table 3:** Association of smoking with all-cause mortality in the study cohort and excluding first two years mortality

	*Study cohort AHR (95% CI)*	*Study cohort excluding mortalities in first 2 years AHR (95% CI)*
**Model 1**		
**Smoking status**		
Non-smoker (Ref.)		
Smoker	**2.42 (1.55–3.76)**	2.58 (1.62–4.09)
**Age** (years)		
25 –34 (Ref.)		
35–44	0.63 (0.28–1.42)	0.59 (0.26–1.36)
45–54	2.51 (1.25–5.02)	2.44 (1.19–5.02)
55–64	6.25 (3.20–12.20)	5.68 (2.83–11.40)
**Model 2**		
**Smoking status**		
Non-smoker (Ref.)		
Smoker	**1.68 (1.03–2.71)**	1.81 (1.10–3.02)
**Age** (years)		
25 –34 (Ref.)		
35–44	0.64 (0.28–1.43)	0.59 (0.26–1.37)
45–54	2.50 (1.25–5.03)	2.43 (1.18–5.02)
55–64	6.32 (3.25–12.31)	5.75 (2.88–11.50)
**Gender**		
Male	2.24 (1.51–3.34)	2.15 (1.41–3.27)
Female (Ref.)		
**Model 3**		
**Smoking status**		
Non-smoker (Ref.)		
Smoker	**1.79 (1.12–2.97)**	1.83 (1.11–3.02)
**Age** (years)		
25 –34 (Ref.)		
35–44	0.56 (0.26–1.21)	0.51 (0.23–1.14)
45–54	1.75 (0.87–53.49)	1.62 (0.79–3.33)
55–64	3.87 (1.94–7.71)	3.27 (1.61–6.65)
**Gender**		
Male	2.37 (1.61–2.50)	2.33 (1.54–3.53)
Female (Ref.)		
**Education level**		
≤Primary	3.36 (1.09–10.38)	3.35 (1.06–10.62)
Secondary	3.14 (1.05–9.33)	2.69 (0.88–8.19)
Tertiary (Ref.)		
**Physical activity**		
Active (Ref.)		
Not active	0.98 (0.62–1.56)	0.94 (0.58–1.53)
**Alcohol drinker**		
Yes	0.87 (0.62–1.56)	0.98 (0.44–2.17)
No (Ref.)		
**Hypercholesterolemia**		
Yes	1.26 (0.79–2.00)	1.34 (0.82–2.20)
No (Ref.)		
**Diabetes mellitus**		
Yes	1.27 (0.91–1.99)	1.14 (0.71–1.47)
No (Ref.)		
**Hypertension**		
Yes	2.13 (1.23–3.57)	2.17 (1.23–3.85)
No (Ref.)		
**Ethnicity**		
Malay	1.39 (0.84–2.30)	1.44 (0.84–2.50)
Chinese	1.26 (0.64–2.44)	1.30 (0.63–2.69)
Other (Ref.)		

AHR: adjusted hazard ratio from multivariable Cox regression analysis. Proportional hazards assumption was met in all models (p>0.05).

## DISCUSSION

Our study showed that smokers had approximately 1.8 times higher risk of all-cause mortality after controlling for multiple confounders. This finding is in line with previous studies in Asian and Western populations. Among them is a meta-analysis of the pooled data of 1002258 individuals involved in 20 prospective cohort studies in six countries (mainland China, Japan, Korea, Singapore, Taiwan, and India), that participated in the Asia Cohort Consortium^20^. Moreover, the prevalence of daily smoking at baseline was 21.1%, which is similar to the rate reported in several national surveys, namely, Global Adult Tobacco Survey Malaysia (GATS-M), National Health and Morbidity Survey (NHMS) 2015 and NHMS 2019. The factors related to smoking found in this study, i.e. high rates among males, ethnic Malays, indigenous people of Sabah and Sarawak, respondents with low education level and residing in rural areas, were also very similar to the aforementioned studies^12,13,29^.

The meta-analysis found a hazard ratio of all-cause mortality of 1.26 (95% CI: 1.17–1.37) for the pre-1920 birth cohort, 1.47 (95% CI: 1.35–1.61) for the 1920s birth cohort, and 1.70 (95% CI: 1.57–1.84) for the cohort born in 1930 or later. A more recent study by Ye et al.^30^ involved 18237 adults in China from 2003–2018 with a total of 579 deaths (7.5% deaths among non-smokers and 11.2% among current smokers) recorded during the 15-year follow-up. In their multivariable analysis which adjusted for drinking, diabetes and hypertension, they found 60% increased risk of all-cause death among current smokers (AHR=1.60; 95% CI: 1.23–2.08) which is similar to that of our study. Similarly, a study by Halipah et al.^21^ among 3353 respondents aged ≥40 years who participated in the Indonesian Family Life Survey (IFLS) found 40.3% prevalence of smoking, and the risk of all-cause of death among current smokers was 48% higher than non-current smokers (HR=1.48; 95% CI: 1.11–1.98).

Similar studies have been conducted in Western nations. The highest hazard ratio was reported by CHANCES (Consortium on Health and Ageing Network of Cohorts in Europe and the United States)^31^. CHANCES was a consortium of 22 population-based cohort studies among older adults in Europe and the United States of America. In the pooled analysis, 99298 deaths were recorded from among the 489056 participants, in which current smokers had 2-fold higher risk of all-cause death compared to non-smokers. The higher HR observed in CHANCES might be due to the older age of its study population (≥60 years); thus, the smoking duration is likely to be longer compared to the current study with ages 25–64 years. Another Western study with a large sample size analyzed data derived from the US National Health and Nutrition and Examination Survey (NHANES) from 1999–2000 to 2013–2014^32^. The study had 30674 participants aged 20–79 years followed-up for an average of 8.3 years, during which 2008 died. A higher risk of mortality among non-smokers (those who smoked on some days) from all causes was observed (AHR=1.50; 95% CI: 1.08–2.08) after adjusting for sociodemographic, lifestyle and chronic diseases (i.e. diabetes, hypertension and dyslipidemia). A study by Akter et al.^33^ was conducted in Japan among the working population who participated in the Japan Epidemiology Collaboration on Occupational Health Study, and included 79114 Japanese workers aged 20–85 years. During the six years follow-up, 252 deaths were reported with adjusted HR for total mortality of 1.49 (95% CI: 1.10–2.01). In summary, these studies showed smokers had increased mortality risk, the magnitude of the association varying slightly due to differences in the sociodemographic profile (e.g. age, ethnicity) of the study population and duration of follow-up.

### Strengths and limitations

Our study has some limitations. First, smoking status was assessed only once at baseline upon the participants’ recruitment and not longitudinally. There is a possibility of respondents’ smoking status changing during the follow-up period (smokers quitting and non-smokers becoming smokers), which may result in an inexact assessment of the relation between smoking and mortality. Second, secondhand smoke (SHS) exposure, a potential confounder, was not measured in this study, which might lead to overestimation of the association between smoking and mortality. Third, the specific actual cause of death among participants cannot be determined.

However, this study has several strengths. First, the results of this study can be generalized to the non-institutionalized population of Malaysia. Second, we also controlled for important potential confounders, including sociodemographic variables such as age, gender and race, and lifestyle risk factors such as physical activity, alcohol consumption, and chronic diseases, which might confound the association between smoking and the risk of death. Third, this study had a relatively long duration of follow-up which allowed better HR estimates. Fourth, the sample size was large with minimal loss to follow-up (1.8%). Fifth, the results of sensitivity analysis we conducted, in which the first two years mortality were excluded, were consistent with the main findings, confirming the validity of the results.

## CONCLUSIONS

Our study indicates that smoking significantly increases the risk of all-cause mortality in Malaysian adults. This study supports previous findings on the risk of smoking on mortality. Therefore, it is important for governments to strengthen their tobacco control policy with participation of all stakeholders and to increase smoking cessation and reduce smoking initiation rates. Smoking cessation services should be made more widely and easily accessible to improve smoking cessation and reduce smoking prevalence. School intervention programs targeting adolescents to reduce early smoking initiation would also reduce smoking prevalence in adults.

## Data Availability

The data supporting this research are available from the authors on reasonable request.

## References

[1] GBD 2019 Tobacco Collaborators (2021). Spatial, temporal, and demographic patterns in prevalence of smoking tobacco use and attributable disease burden in 204 countries and territories, 1990-2019: a systematic analysis from the Global Burden of Disease Study 2019. Lancet.

[2] World Health Organization Tobacco.

[3] World Health Organization (2018). WHO Global Report on Trends in Prevalence of Tobacco Smoking 2000-2025.

[4] Oberg M, Jaakkola MS, Woodward A, Peruga A, Prüss-Ustün A (2011). Worldwide burden of disease from exposure to second-hand smoke: a retrospective analysis of data from 192 countries. Lancet.

[5] Kulhánová I, Forman D, Vignat J (2020). Tobacco-related cancers in Europe: The scale of the epidemic in 2018. Eur J Cancer.

[6] Bialous SA, Sarna L (2017). Lung Cancer and Tobacco: What Is New?. Nurs Clin North Am.

[7] Ministry of Health Malaysia - Institute for Public Health (2015). National Health and Morbidity Survey 2015. Healthcare Demand; vol 20.

[8] Aljunid SM (2007). Health care cost of smoking in Malaysia.

[9] Ministry of Health Malaysia Malaysia National Strategic Plan for Tobacco Control 2015-2020. Pelan Strategik Kebangsaan Bagi Kawalan Tembakau 2015-2020.

[10] Ministry of Health Malaysia - Institute of Public Health (1997). National Health and Morbidity Survey 1996. Smoking; vol 15.

[11] Ministry of Health Malaysia - Institute of Public Health (2008). The Third National Health and Morbidity Survey 2006 (NHMS III): Smoking.

[12] Ministry of Health Malaysia - Institute of Public Health (2012). Global Adult Tobacco Survey (GATS): Malaysia 2011.

[13] Lim KH, Teh CH, Pan S (2018). Prevalence and factors associated with smoking among adults in Malaysia: Findings from the National Health and Morbidity Survey (NHMS) 2015. Tob Induc Dis.

[14] Reisi M, Javadzade SH, Shahnazi H, Sharifirad G, Charkazi A, Moodi M (2014). Factors affecting cigarette smoking based on health-belief model structures in pre-university students in Isfahan, Iran. J Educ Health Promot.

[15] GBD 2015 Tobacco Collaborators (2017). Smoking prevalence and attributable disease burden in 195 countries and territories, 1990-2015: a systematic analysis from the Global Burden of Disease Study 2015. Lancet.

[16] Ezzati M, Henley SJ, Thun MJ, Lopez AD (2005). Role of smoking in global and regional cardiovascular mortality. Circulation.

[17] Tachfouti N, Raherison C, Najdi A (2014). Smoking-attributable mortality in Morocco: results of a prevalence-based study in Casablanca. Arch Public Health.

[18] Gu D, Kelly TN, Wu X (2009). Mortality attributable to smoking in China. N Engl J Med.

[19] Corrêa PC, Barreto SM, Passos VM (2009). Smoking-attributable mortality and years of potential life lost in 16 Brazilian capitals, 2003: a prevalence-based study. BMC Public Health.

[20] Yang JJ, Yu D, Wen W (2019). Tobacco Smoking and Mortality in Asia: A Pooled Meta-analysis. JAMA Netw Open.

[21] Holipah H, Sulistomo HW, Maharani A (2020). Tobacco smoking and risk of all-cause mortality in Indonesia. PLoS One.

[22] Banks E, Joshy G, Weber MF (2015). Tobacco smoking and all-cause mortality in a large Australian cohort study: Findings from a mature epidemic with current low smoking prevalence. BMC Med.

[23] Doll R, Peto R, Boreham J, Sutherland I (2004). Mortality in relation to smoking: 50 Years’ observations on male British doctors. BMJ.

[24] Thun MJ, Carter BD, Feskanich D (2013). 50-Year trends in smoking-related mortality in the United States. N Engl J Med.

[25] Ministry of Health Malaysia (2006). Malaysia NCD Surveillance 2006: NCD Risk Factors in Malaysia.

[26] World Health Organization Global Physical Activity Questionnaire (GPAQ): Analysis Guide.

[27] Chobanian AV, Bakris GL, Black HR (2003). Seventh Report of the Joint National Committee on Prevention, Detection, Evaluation, and Treatment of High Blood Pressure. Hypertension.

[28] World Health Organization (1999). Definition, Diagnosis and Classification of Diabetes Mellitus and its Complications: Report of a WHO Consultation. Part 1: Diagnosis and Classification of Diabetes Mellitus.

[29] Ministry of Health Malaysia - Institute for Public Health (2020). National Health and Morbidity Survey (NHMS) 2019. NCDs – Non-Communicable Diseases: Risk Factors and other Health Problems; vol. 1.

[30] Ye L, Yang J, Li J (2021). Cigarette smoking and all-cause mortality in rural Chinese male adults: 15-year follow-up of the Anqing cohort study. BMC Public Health.

[31] Müezzinler A, Mons U, Gellert C (2015). Smoking and All-cause Mortality in Older Adults: Results From the CHANCES Consortium. Am J Prev Med.

[32] Zhu D, Zhao G, Wang X (2021). Association of smoking and smoking cessation with overall and cause-specific mortality. Am J Prev Med.

[33] Akter S, Nakagawa T, Honda T (2018). Smoking, Smoking Cessation, and Risk of Mortality in a Japanese Working Population - Japan Epidemiology Collaboration on Occupational Health Study. Circ J.

