# Supplementary material for: Assessment of association between smoking and all-cause mortality among Malaysian adult population: Findings from a retrospective cohort study
Source: Tob Induc Dis. 2022 May 31;20:50. doi: 10.18332/tid/147656 (PMC9150905; doi:10.18332/tid/147656)
Supplement: Supplementary file 1 [file TID-20-50-s1.pdf]

Supplementary Table. Association of smoking with all-cause mortality for male participants in the whole study cohort and excluding first two years mortality

	Whole cohort AHR (95% CI)	Proportional hazards test p value	Cohort excluding mortalities in first 2 years AHR (95% CI)	Proportional hazards test p value
Model 3				
Smoking status				
Non-smoker	Ref.	0.007	Ref.	0.023
Smoker	1.63(1.01-2.63)		1.79(1.07-2.97)	
Age group				
25-34	Ref.		Ref.	
35-44	0.28(0.01-0.77)		0.23(0.08-0.67)	
45-54	1.85(0.83-4.14)		1.73(0.75-4.00)	
55-64	4.29(1.93-9.54)		3.60(1.58-8.21)	
Gender				
Male	2.37(1.61-2.50)		2.33(1.54-3.53)	
Female	Ref.		Ref.	
Education level				
Primary	3.61(1.34-9.75)		3.55(1.29-9.75)	
Secondary	3.25(1.24-8.51)		2.88(1.08-7.65)	
Tertiary	Ref.		Ref.	
Physical activity				
Active	Ref.		Ref.	
Not active	0.94(0.66-1.34)		092(0.63-1.35)	
Alcohol drinker				
Yes	1.14(0.59-2.19)		1.26(0.61-2.58)	
No	Ref.		Ref.	
Hypercholesterolemia				
Yes	1.13(0.78-1.64)		1.17(0.79-1.73)	
No	Ref.		Ref.	
Diabetes mellitus				
Yes	1.07(0.75-1.53)		1.01(0.70-1.42)	
No	Ref.		Ref.	
Hypertension				
Yes	2.50(1.59-3.85)		2.56(1.59-4.17)	
No	Ref.		Ref.	

Ethnicity		
Malay	1.90(0.99-3.62)	2.04(1.01-4.12)
Chinese	1.47(0.84-2.30)	1.68(0.68-4.12)
Indian	1.42(0.59-3.42)	1.48(0.57-3.83)
Others	Ref.	Ref.

© 2022 Lim K.H. et al.
